# Transection injury differentially alters the proteome of the human sural nerve

**DOI:** 10.1371/journal.pone.0260998

**Published:** 2022-11-23

**Authors:** Monica J. Chau, Jorge E. Quintero, Eric Blalock, Stephanie Byrum, Samuel G. Mackintosh, Christopher Samaan, Greg A. Gerhardt, Craig G. van Horne

**Affiliations:** 1 Brain Restoration Center, College of Medicine, University of Kentucky, Lexington, KY, United States of America; 2 Department of Neurosurgery, College of Medicine, University of Kentucky, Lexington, KY, United States of America; 3 Department of Neuroscience, College of Medicine, University of Kentucky, Lexington, KY, United States of America; 4 Department of Pharmacology and Nutritional Sciences, College of Medicine, University of Kentucky, Lexington, KY, United States of America; 5 Department of Biochemistry and Molecular Biology, University of Arkansas for Medical Sciences, Little Rock, AR, United States of America; 6 Department of Neurology, College of Medicine, University of Kentucky, Lexington, KY, United States of America; University of Miami, UNITED STATES

## Abstract

Regeneration after severe peripheral nerve injury is often poor. Knowledge of human nerve regeneration and the growth microenvironment is greatly lacking. We aimed to identify the regenerative proteins in human peripheral nerve by comparing the proteome before and after a transection injury. In a unique study design, we collected closely matched samples of naïve and injured sural nerve. Naïve and injured (two weeks after injury) samples were analyzed using mass spectrometry and immunoassays. We found significantly altered levels following the nerve injury. Mass spectrometry revealed that injury samples had 568 proteins significantly upregulated and 471 significantly downregulated compared to naïve samples (q-value ≤ 0.05 and Z ≥ |2| (log2)). We used Gene Ontology (GO) pathway overrepresentation analysis to highlight groups of proteins that were significantly upregulated or downregulated with injury-induced degeneration and regeneration. Significant protein changes in key pathways were identified including growth factor levels, Schwann cell de-differentiation, myelination downregulation, epithelial-mesenchymal transition (EMT), and axonal regeneration pathways. The proteomes of the uninjured nerve compared to the degenerating/regenerating nerve may reveal biomarkers to aid in the development of repair strategies such as infusing supplemental trophic factors and in monitoring neural tissue regeneration.

## Introduction

Twenty million Americans are affected by peripheral nerve injury [1]. In retrospective studies of peripheral nerve injuries between 1989 and 2014, most are from traumatic incidents and are upper limb injuries [2,3]. The top causes were vehicular accidents (46.4%), penetrating trauma (23.9%), falls (10.9%), and gunshot wounds (6.6%) [2,3]. Unfortunately, peripheral nerve repair is slow and often incomplete. The sooner the nerve can be repaired after a transection, the better the outcome as the regenerative processes can attempt to reconnect the ends. Even under optimal nerve repair circumstances, axonal regeneration is still slow at 1–2 mm/day [4] and may be incomplete. A meta-analysis of reconstructing the median and ulnar nerves showed that only 51.6% had satisfactory motor recovery and 42.6% reporting satisfactory sensory recovery [5]. After severe peripheral nerve injury, there is poor regeneration and functional recovery. Understanding the transcriptome [6], proteome, and the mechanisms that underlie peripheral nerve regeneration is essential to further develop peripheral nervous system (PNS) regenerative therapies and to improve clinical outcomes. Even though there is a great deal that is known about peripheral nerve regeneration and the growth-permissive microenvironment in animal models, results on human nerves is sparse and inconclusive [7,8]. Multiple components contribute to peripheral nerve repair after injury notably Schwann cells [9–12], fibroblasts, endothelial cells, and immune cells such as macrophages [13–15]. An injured peripheral nerve undergoes three main processes for recovery and reestablishment of a functional connection with its distal end [16]. First, within 24–48 hours after transection injury of the nerve, Wallerian degeneration starts in which the distal end of the transection undergoes axonal degeneration. Afterward, the myelin is degraded as macrophages infiltrate this area. Secondly, axonal regeneration begins, leading to the third step of end-organ re-innervation. Macrophages and Schwann cells play major roles in mediating degeneration and regeneration.

Animal models of peripheral nerve regeneration demonstrate that Schwann cells have remarkable plasticity. They rapidly adapt to injury through extensive cellular reprogramming that transforms their myelinating phenotype into a reparative phenotype [17,18]. Studies have delineated two phases of the Schwann cell injury response: 1) the clearing of myelin [9] and de-differentiation of the cell’s myelinating phenotype and 2) the maturation into the repair Schwann cell phenotype in which the cells promote survival by releasing neuroprotective factors and increasing anti-apoptosis factors. Schwann cells upregulate and release a whole host of neurotrophic and cell survival factors including glial cell-derived neurotrophic factor (GDNF) [19,20], neurotrophin-3 (NT-3), nerve growth factor (NGF) [21,22], brain derived neurotrophic factor (BDNF) [23], vascular endothelial growth factor (VEGF), erythropoietin (EPO) [24], pleiotrophin, and N-cadherin [11,12,25–27].

Human peripheral nerve injury studies are typically limited to collecting nerve tissues *after* an injury (*e*.*g*. after a trauma) while obtaining healthy, uninjured nerve beforehand has not been as feasible [28]. As such, many aspects of human peripheral nerve regeneration are unknown including the profiles of protein upregulation and downregulation. Understanding these changes would help optimize treatment discovery and clinical management strategies for improving PNS repair outcomes.

To address these gaps in knowledge, we performed a comprehensive proteomic analysis to include more than 5000 of the most abundant proteins found in peripheral (sural) nerve *before and after* a transection injury. We hypothesized that transecting a peripheral nerve and leaving it in place within the participant for two weeks would increase synthesis in the pathways associated with nerve regeneration. Thus, this proteomic profile would provide a database to identify new targets for improving regeneration outcomes.

## Materials and methods

### Research subjects

The University of Kentucky’s Institutional Review Board approved this study and the participants provided written informed consent before participating in the study. Sural nerve samples were being collected as part of a clinical trial examining the reparative capabilities of peripheral nerve tissue [29]. Participants were diagnosed with idiopathic Parkinson’s disease (PD). Nine were female, 17 were male (assigned birth sex). Their ages ranged from 50–70 years old (mean 61 years old). Years diagnosed with PD ranged from 2–17 years (mean 10 years). Tables 1 and 2 list participant demographics from both types of proteomic analyses (mass spectrometry and targeted immunoassays). Note that there were three participants whose tissues were used in both mass spectrometry and targeted immunoassay.

**Table 1:** Mass spectrometry participant demographics.

Participant	Age (years)	Years diagnosed	Assigned Birth Sex
1	70	7	Male
2	70	12	Female
3	63	11	Male
4	69	9	Male
5	66	2	Female
6	66	16	Male
7	52	6	Female
8	60	6	Male
9	57	10	Male
10	60	7	Female
11	50	8	Male
12	61	9	Male
13	61	8	Male
14	65	6	Male

Table 1. Mass spectrometry study participant demographics listed include age, years diagnosed with PD, and assigned birth sex.

**Table 2:** Targeted immunoassays participant demographics.

Participant	Age (years)	Years diagnosed	Assigned Birth Sex
15	51	17	Male
7	52	6	Female
17	54	16	Male
18	56	10	Male
19	58	12	Female
20	60	13	Female
21	61	15	Female
13	61	8	Male
23	62	4	Male
24	62	7	Male
25	64	16	Male
26	65	8	Male
14	65	6	Male
28	66	9	Female
29	69	8	Female

Table 2. Immunoassay study participant demographics listed include age, years diagnosed with PD, and assigned birth sex.

### Peripheral nerve transection and tissue collection

**Fig 1:**
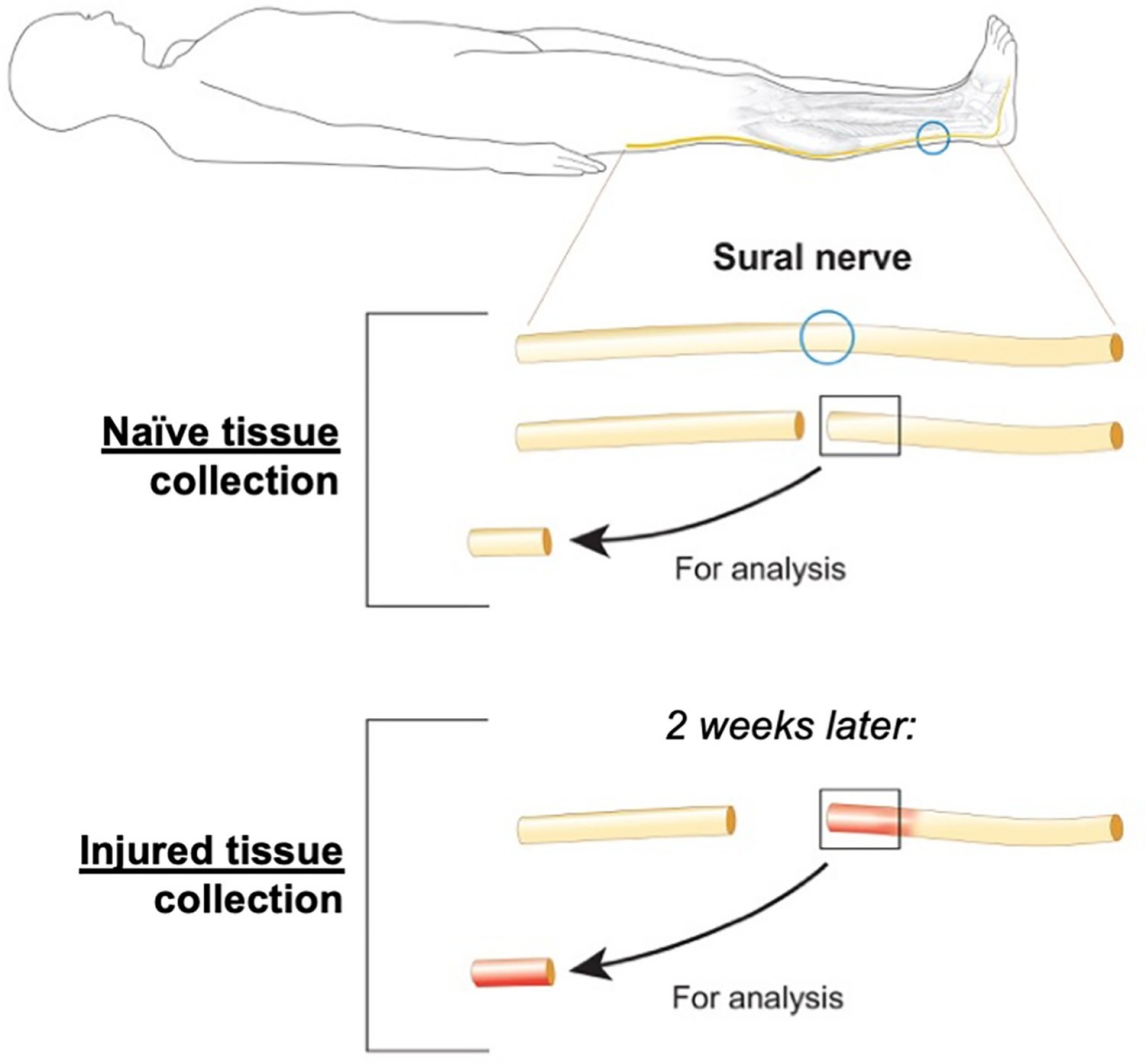
Study overview. Schematic to illustrate the timing and location of the sural nerve injury and tissue collection.

Our approach in transecting the peripheral nerve and collecting tissue has been previously described [6,29,30]. Fig 1 illustrates the timing of the transection and tissue collections. Briefly, sural nerve samples were collected at two different time points: a naïve/non-injured sample collected during the first surgery followed by a post-transection (injured) sample from the second surgery two weeks later. To collect the naive tissue, the neurosurgeon identified the neurovascular bundle containing the sural nerve in the ankle. Two sutures were tied around the nerve, 1 cm apart. The section of nerve just proximal to the sutures was transected, and a 1-cm segment was excised for analysis. Individual nerve fascicles were separated, snap-frozen, and stored for assays as in Welleford et al. [6].

To collect post-transection tissue for both implantation in the clinical trial and proteomic analysis, the ankle incision was reopened after two weeks, suture markers were located, and a new 1–2 cm segment was collected from the distal nerve stump for analyses and implantation as part of the trial design. Individual nerve fascicles were separated, snap-frozen, and stored for assays [6].

### Proteomics analyses

#### Mass spectrometry proteomics

**Fig 2:**
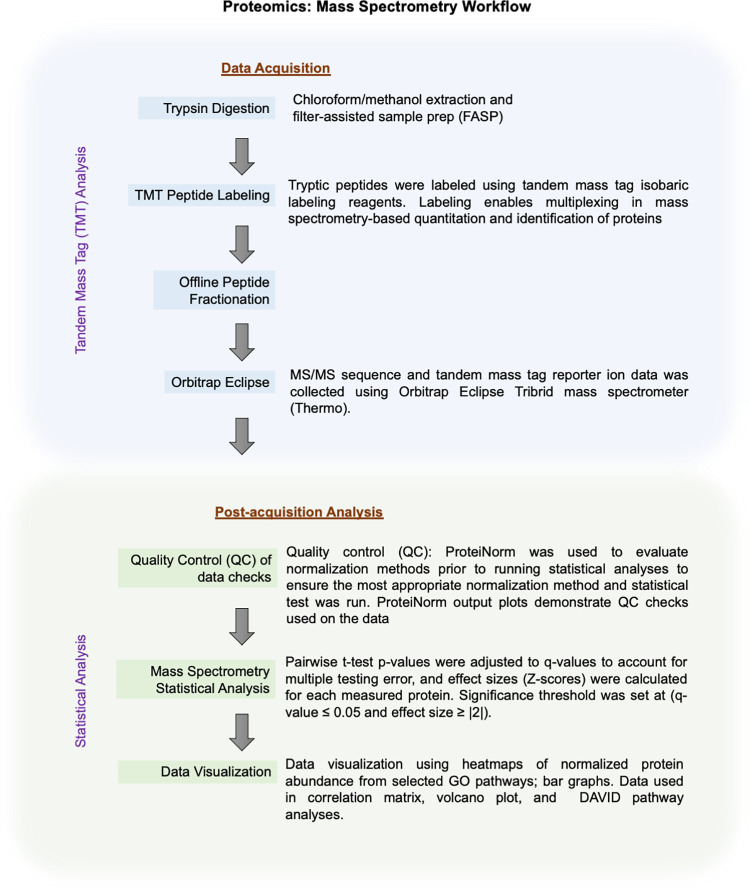
Mass spectrometry workflow. Flowchart to illustrate the steps of mass spectrometry (data acquisition), and the statistical analysis (post-acquisition analysis).

Tandem Mass Tag (TMT) analysis was used which is a global, unbiased method. Fig 2 illustrates the workflow steps used in mass spectrometry and post-acquisition statistical analysis. As many proteins/peptides as possible were detected and searched against a species-specific database. Total protein was isolated from peripheral nerve tissue samples. Protein samples were prepared for mass spectrometric analysis, and MS/MS sequence and TMT reporter ion data was collected using a state-of-the-art Orbitrap Eclipse instrument. Quantitative analysis was performed to obtain a comprehensive proteomic profile.

Proteins were identified and quantified with the TMT MS3 reporter ion intensities using MaxQuant (version 1.6.17.0) against the UniprotKB Homo sapiens database (July 2020) and visualized with Scaffold using 1% false discovery thresholds at both the protein and peptide level. The data was checked for quality and normalized using an in-house ProteiNorm Shiny app, a tool for a systematic evaluation of normalization methods, imputation of missing values and comparisons of different differential abundance methods. Missing values were filtered so each sample had to have a protein with an expressed abundance and the data was normalized using Cyclic Loess [31].

CME bHPLC TMT Methods–Orbitrap Eclipse:

Proteins were reduced, alkylated, and purified by chloroform/methanol extraction prior to digestion with sequencing grade modified porcine trypsin (Promega) (Fig 2). Tryptic peptides were labeled using TMT isobaric labeling reagents (Thermo) following the manufacturer’s instructions and combined into three 11-plex sample groups with a pooled reference sample in each group. Each labeled peptide multiplex was separated into 46 fractions on a 100 x 1.0 mm Acquity BEH C18 column using an UltiMate 3000 UHPLC system (Thermo) with a 50 min gradient from 99:1 to 60:40 buffer A:B ratio under basic pH conditions, and then consolidated into 18 super-fractions. Each super-fraction was then further separated by reverse phase XSelect CSH C18 2.5 μm resin on an in-line 150 x 0.075 mm column using an UltiMate 3000 RSLCnano system (Thermo). Peptides were eluted using a 60 min gradient from 98:2 to 60:40 buffer A:B ratio (buffer A consists of 0.1% formic acid, 0.5% acetonitrile; buffer B consists of 0.1% formic acid, 99.9% acetonitrile; and both buffers were adjusted to pH 10 with ammonium hydroxide for offline separation). Eluted peptides were ionized by electrospray (2.2 kV) followed by mass spectrometric analysis on an Orbitrap Eclipse Tribrid mass spectrometer (Thermo) using multi-notch MS3 parameters with real-time search enabled. MS data were acquired using the FTMS analyzer in top-speed profile mode at a resolution of 120,000 over a range of 375 to 1500 m/z. Following CID activation with normalized collision energy of 35.0, MS/MS data were acquired using the ion trap analyzer in centroid mode and normal mass range. Using synchronous precursor selection, up to 10 MS/MS precursors were selected for HCD activation with normalized collision energy of 65.0, followed by acquisition of MS3 reporter ion data using the FTMS analyzer in profile mode at a resolution of 50,000 over a range of 100–500 m/z.

**Fig 3:**
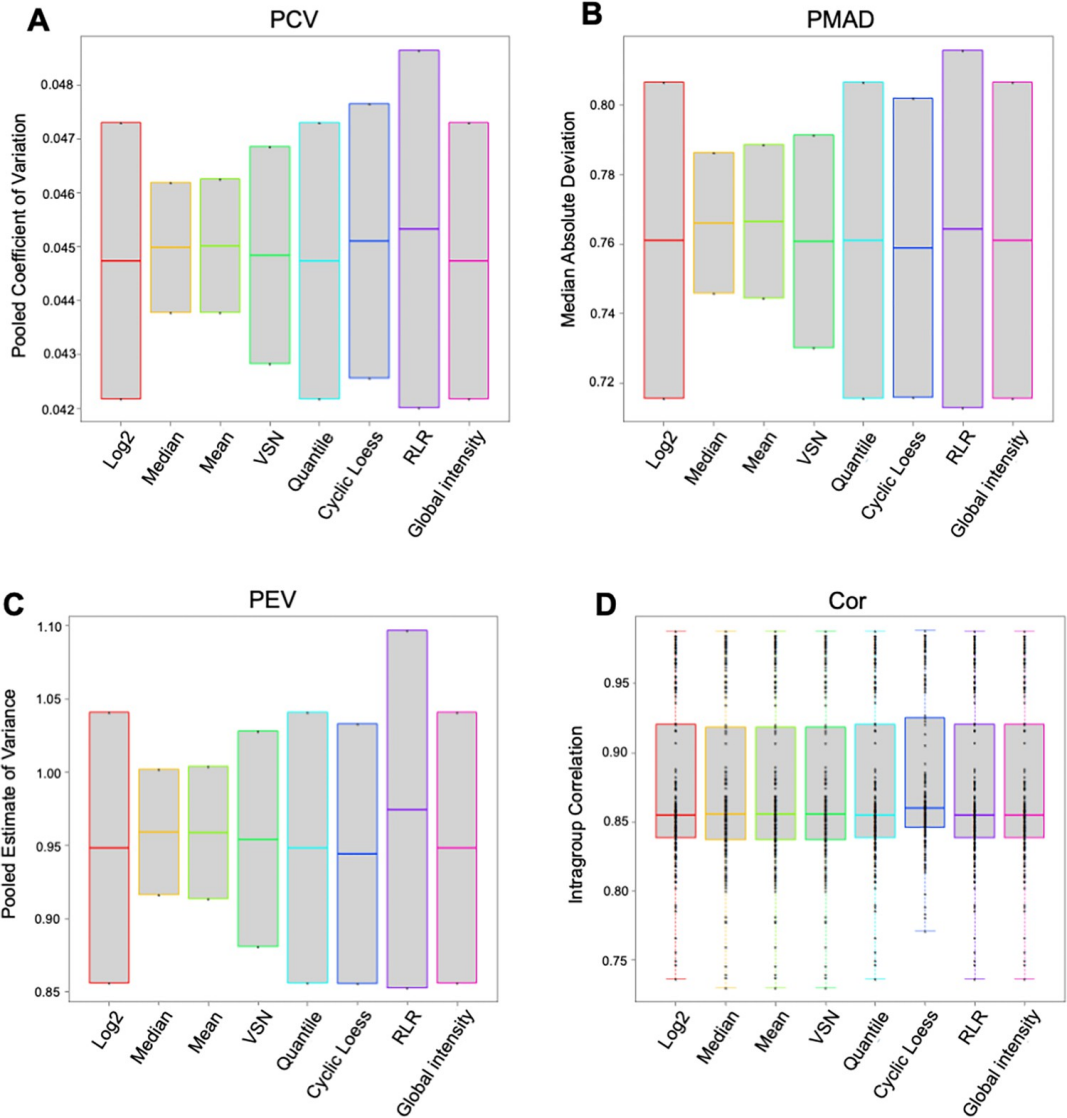
Quality control checks of mass spectrometry data. **A-C.** Pooled coefficient of variance (PCV), pooled median absolute deviation (PMAD), and pooled estimate of variance (PEV) were all small in these analyses. Small numbers here are ideal since they show low variation of the sample replicates. D. Cor graph compares the pairwise sample correlations of the treatment groups of the normalization methods. The high intragroup correlation values here are desirable since they show high correlation of the replicates within a treatment group.

Quality Control Checks for Mass Spectrometry Data:

Each data set had its own characteristics so we performed quality control (QC) checks of data prior to running statistical analyses to ensure the most appropriate normalization method and statistical test was run (Fig 3). ProteiNorm output plots demonstrate QC checks used on the data [32] (Fig 3). ProteiNorm was developed by [32], as a solution for “systemic bias from unknown sources” in large protein data sets allowing to prevent erroneous conclusions.

ProteiNorm is a tool used to systematically evaluate normalization methods in mass spectrometry [32]. This includes linear regression, local regression, total intensity, average intensity, median intensity, VSN, and quantile normalization [32]. Each of these methods is evaluated with comparisons of its pooled coefficient of variance (PCV), pooled estimate of variance (PEV), and pooled median absolute deviation (PMAD) (Fig 3). PCV, PEV, and PMAD (Fig 3A–3C) from our analyses here were all small values. Small numbers here are desirable since they show low variation of the sample replicates. The Cor graph (Fig 3D) is a comparison of the pairwise sample correlations of the treatment groups of the normalization methods. High intragroup correlation values are desirable [32]. The MS3 reporter ion intensities were evaluated for the best normalization method using ProteiNorm [32]. Cyclic Loess normalization performed the best as shown in Fig 3 as having comparable intragroup variation and the highest intragroup correlation.

Pre-statistical procedures:

Mass spectrometry quantification (log 2 scale, Cyclic Loess normalized) data included 5,573 rows for different proteins mapped back to their gene symbol representations for 28 paired (naïve and injured) samples from 14 subjects.

‘0’ values:

Seventeen percent of the data space (# rows * # samples = 156,044) was occupied by ‘0’ values. These ‘0’ values could represent low levels (below the detection threshold), no levels, or technical error [33]. In proteomic data, ‘0’s are considered missing-not-at-random’ (MNAR), and various strategies have been proposed to address this property of the data [34]. Here, we used a two-step procedure to address the ‘0’s. First, the number of ‘0’s on each row were quantified and rows with < 20 / 28 ‘0’s (i.e., at least seven reliable signal intensity measures on the row) were retained for further analysis (5,508 rows of data were retained). Second, within this filtered data set, the remaining ‘0’s were treated as missing values.

Annotation:

Fourteen protein symbols with date-like names (from the MAR, MARC, SEP, and SEPT families) were updated to reflect newer non-date-like protein symbols (e.g., SEP15 was changed to SELENOF) to avoid their accidental conversion to date codes in spreadsheet programs. Additionally, 434 instances of ‘repeated’ annotations (that is, more than one row reporting the same gene symbol) were identified. Among these, the row with the highest average signal level was retained for analysis, resulting in a final data set of (28 samples * 5074) uniquely protein symbol mapped proteins with reliable signal intensities.

Mass Spectrometry Statistical Analysis:

Prior to performing statistical analysis, PCA and correlation matrices (not shown) both indicated the presence of a batch effect (batch 1 participants: 13,14, 8, 12, 11; batch 2 participants: 10, 2, 4, 3, 5; batch 3- subjects: 1, 6, 7, 9). Within each batch, uninjured and injured samples from the same subjects were tightly clustered. To remove the batch effect, each protein measure was standardized across all subjects’ pre and post-injury measures within each batch, and then the batch-standardized data was recombined from all batches for further analysis. Batch standardized data was analyzed by pairwise t-test adjusted for multiple testing error to calculate the q-value [35], as well as by Z-scored effect size. The final results contained the Z-scored effect size, unadjusted p-values, and FDR adjusted q-values. Significance threshold is a Z ≥ |2| (on log2 scale) and q ≤ 0.05.

Volcano plot:

For the total of 5074 proteins included in the analysis, effect size (x axis) were plotted against the log 10 p-values (y axis) for each protein. Highly stringent p-value (≤ 1E-10) and Z-score (≥ |16|; or as expressed on the log 2 scale, ≥ |4|) cutoffs were used to highlight proteins with the largest and most significant changes of the entire dataset.

DAVID pathway analysis:

DAVID pathway analysis was performed using the GO database. The GO database consists of three ontologies, one for cellular component, one for molecular function, and one for biological process [36,37]. The online software tool, DAVID works by grouping together genes based on similar functions. When given a list of differentially expressed genes or proteins, DAVID uses a fuzzy clustering algorithm. It takes information in its knowledge base on genes/proteins and their functional associations. It then groups genes together that are statistically significant with their association to a group of similar functional categories. We report the overrepresentation adjusted p-value (adjusted for multiple testing).

GO pathway analyses and heatmap generation:

Selected GO pathways of interest that were specifically related to peripheral nerve injury and regenerative processes were analyzed aligning with our group’s transcriptome analyses [6]. The visualized mapped proteins represent the significantly differentially expressed proteins from each pathway. The proteins that corresponded to the GO pathways that were significantly upregulated or downregulated based off the effect size were graphed. Heatmaps were created in Microsoft Excel using conditional formatting to depict the range of effect sizes using a red-white-blue gradient.

#### Immunoassays for proteins of interest: ELISA and Luminex

Certain proteins of interest were not detected with mass spectrometry because they were not in high enough abundance. For these proteins of interest, such as growth factors, we used enzyme-linked immunosorbent assay (ELISA) and multiplex Luminex® immunoassays (Cincinnati Children’s Hospital Flow Cytometry Core) to detect their change after injury. We compared naïve and injured tissues, from 15 participants, three of these participants’ tissues were also used in mass spectrometry analysis (see Tables 1 and 2).

Analyte concentrations in the sample supernatants were determined by ELISA according manufacturer’s protocol. The ELISA and antibody sources and dilutions were: Cerebral dopamine neurotrophic factor (CDNF) (Abcam, Cambridge, MA), samples were diluted 1:100. Nuclear factor erythroid 2-related factor 2 (NRF2) (ThermoFisher Scientific, Carlsbad, CA), samples diluted 1:2. Nerve Growth Factor Receptor (NGFR) (ThermoFisher Scientific, Carlsbad, CA), samples were diluted 1:2. B cell lymphoma 6 (BCL-6) (MyBiosource, San Diego, CA), samples were neat. EPO concentrations in the sample supernatants were determined by using Milliplex^®^ Multiplex kits (MilliporeSigma, Darmstadt, Germany) and BDNF, Beta-NGF, Platelet-Derived Growth Factor (PDGF)-AA, VEGF, GDNF, NT-3, PDGF-BB, PDGF-AB were determined by Human Magnetic Luminex Assays (R&D Systems, Minneapolis, MN) according to manufacturer’s protocol.

Analysis:

For each protein analyzed with the immunoassay, the mean difference between the naïve and injured samples and the 95% confidence interval (Excel, Microsoft) is reported.

#### Availability of data and materials

Datasets are available and listed with the DOI: https://doi.org/10.13023/cbz6-ea76.

## Results

### A clear proteomic distinction between naïve and injured nerve

**Fig 4:**
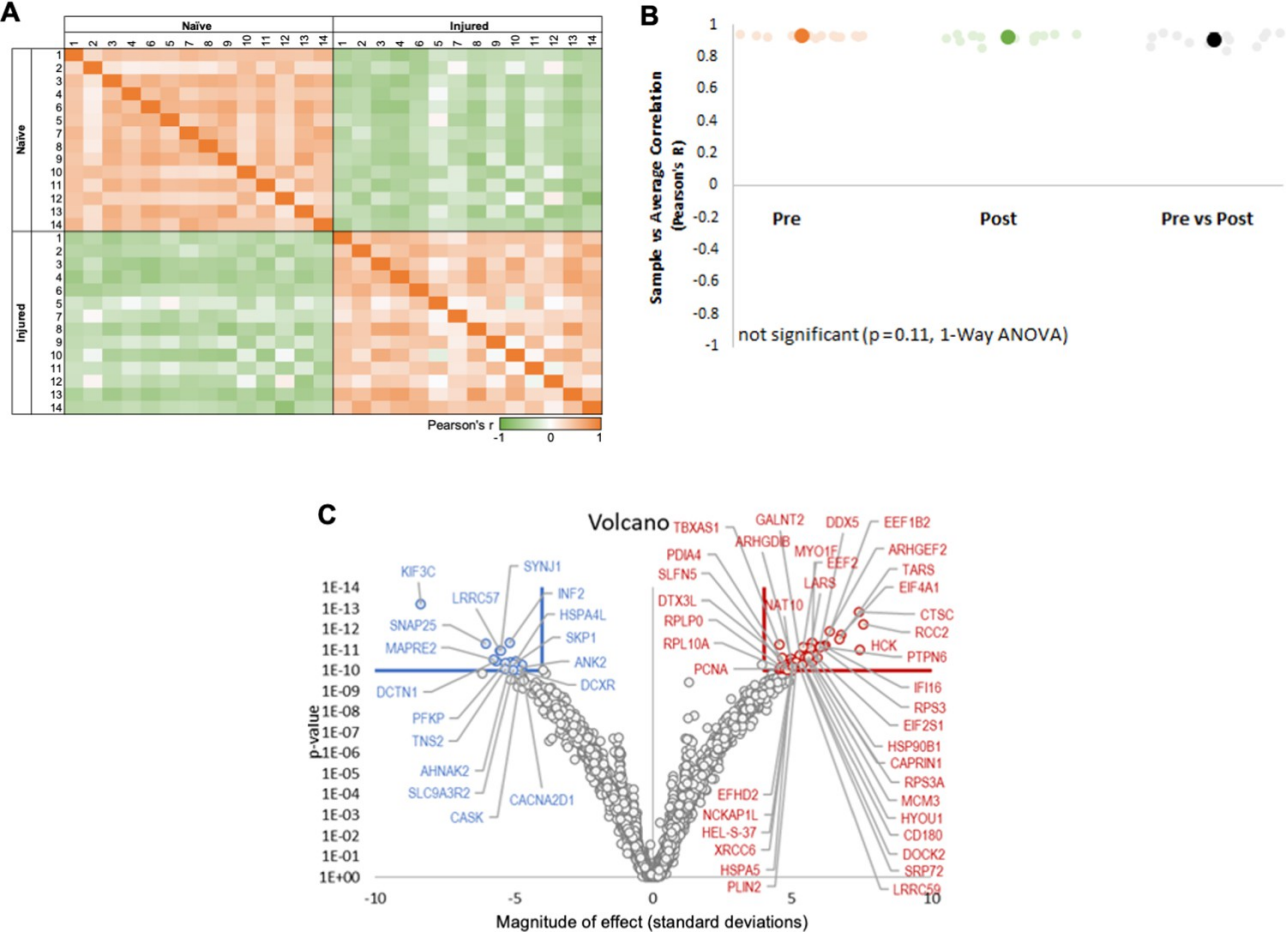
Clear Proteomic Changes Between Naïve and Injured Nerve. **A.** Overall proteomic expression was altered after nerve injury based on Pearson *r* correlation. The Pearson’s correlation R-value for each sample, correlated with every other sample is displayed in a correlation matrix of naïve and injured samples (matrix scale orange R = 1: Perfect correlation, green R = -1: Perfect anti-correlation). **B.** Lin’s CCC demonstrated strong homogeneity among samples within the pre- and post-injury groups **C.** The volcano plot shows proteins that were downregulated (blue) and upregulated (red) after injury. For the volcano plot, the p-value cutoff was 1E-10 and the Z (Log2(FC)) cutoff (log2 scale) was |4|.

Normalized protein levels from the naive and injured treatment conditions were positively correlated within treatment and negatively correlated across treatment (Fig 4A) based on Pearson’s *r* correlation, indicating a strong injury-based influence on the proteome. The Pearson’s correlation R-value for each sample, correlated with every other sample is displayed in a correlation matrix.

We calculated Lin’s Concordance Correlation Coefficient (CCC), which quantifies degree of similarity among different samples. Results indicated strong positive, and highly similar profiles, from each sample compared to the average of all samples within either the pre-injury or post-injury groups (Fig 2B). CCCs in Pre-injury (CCC = 0.936 +/- 0.0065) and Post-injury (CCC = 0.93 +/- 0.012) conditions were greater than the 0.8 typically interpreted as showing strong similarity, and thus indicates generally strong homogeneity among samples within the pre- and post-injury groups.

Among the most significant protein changes of the *entire* mass spectrometry dataset, the volcano plot shows 17 proteins that were downregulated (blue) and 41 upregulated proteins (red) after injury (Fig 4C). The volcano plot cutoffs were more stringent than in each individual GO heatmap to highlight the most upregulated and downregulated proteins of the entire data set. For the volcano plot, the p-value cutoff is 1E-10 and the Z cutoff (on log2 scale) is |4|.

### Global functional analysis

We used DAVID pathway analysis to perform functional over-representation analysis on the GO set of term annotations [37]. We show the top significantly upregulated and downregulated GO pathways after nerve injury and report their overrepresentation adjusted p-value (Tables 3 and 4). The analyses showed that 8 of the 20 most significantly upregulated pathways (Table 3) were related to gene expression through translation (8 of the 20 total GO terms: ribonucleoprotein complex, ribonucleoprotein complex biogenesis, regulation of translation, RNA processing, translational initiation, regulation of gene expression, aminoacyl-tRNA synthetase multienzyme complex, ribosome assembly).

**Table 3:** The top significantly upregulated (GO) pathways after injury.

GO ID	Increased two weeks after injury	#	adj. p-val
GO:0003723	RNA binding	241	1.65E-37
GO:1990904	ribonucleoprotein complex	158	4.44E-34
GO:0022613	ribonucleoprotein complex biogenesis	76	2.33E-13
GO:0005783	endoplasmic reticulum	163	7.76E-13
GO:0006417	regulation of translation	62	4.03E-10
GO:0071013	catalytic step 2 spliceosome	30	8.64E-09
GO:0006396	RNA processing	95	2.04E-08
GO:0034622	cellular macromolecular complex assembly	113	2.40E-07
GO:0022624	proteasome accessory complex	15	3.95E-07
GO:0006413	translational initiation	29	1.58E-06
GO:0065004	protein-DNA complex assembly	28	2.46E-06
GO:0017101	aminoacyl-tRNA synthetase multienzyme complex	10	5.89E-06
GO:0006888	ER to Golgi vesicle-mediated transport	27	2.19E-05
GO:0010468	regulation of gene expression	180	4.92E-05
GO:0030055	cell-substrate junction	66	1.28E-04
GO:0005793	endoplasmic reticulum-Golgi intermediate compartment	25	1.28E-04
GO:0032200	telomere organization	27	1.66E-04
GO:0060205	cytoplasmic vesicle lumen	52	2.16E-04
GO:1903312	negative regulation of mRNA metabolic process	14	7.60E-04
GO:0042255	ribosome assembly	16	9.77E-04

Table 3. The top significantly upregulated GO Pathways after nerve injury, the # of proteins in each pathway that were found to be significant, and their overrepresentation adjusted p-values.

**Table 4:** The top significantly downregulated (GO) pathways after injury.

GO ID	Decreased two weeks after injury	#	adj. p-val
GO:0097458	neuron part	129	7.94E-17
GO:0030054	cell junction	100	2.57E-11
GO:0099512	supramolecular fiber	74	3.35E-09
GO:0044456	synapse part	74	1.78E-07
GO:0015630	microtubule cytoskeleton	82	3.94E-07
GO:1903561	extracellular vesicle	170	8.03E-07
GO:0097060	synaptic membrane	29	9.09E-06
GO:0005875	microtubule associated complex	20	1.32E-05
GO:0034330	cell junction organization	35	5.19E-05
GO:0007399	nervous system development	119	5.19E-05
GO:0007010	cytoskeleton organization	94	5.19E-05
GO:0007267	cell-cell signaling	70	5.27E-05
GO:0003008	system process	80	5.40E-05
GO:0016010	dystrophin-associated glycoprotein complex	8	7.24E-05
GO:0006928	movement of cell or subcellular component	109	1.06E-04
GO:0001664	G-protein coupled receptor binding	24	2.33E-04

Table 4. The top significantly downregulated GO Pathways after nerve injury, the # of proteins in each pathway that were found to be significant, and their overrepresentation adjusted p-values.

The top significantly downregulated pathways (Table 4) were related to cytoskeletal structure or organization (5 of the 16 total GO terms were: supramolecular fiber, microtubule cytoskeleton, microtubule associated complex, cytoskeleton organization, dystrophin-associated glycoprotein complex) and neurons and synapses (4 out of the 16 total GO terms were: neuron part, synapse part, synaptic membrane, nervous system development). In Tables 3 and 4, we report the # of proteins in each pathway that were found to be significant (second to last column).

### Significant protein changes shown in GO pathways and heatmaps

After processing, mass spectrometry analysis of naïve and injured sural nerve returned a total of 5074 proteins. When comparing naïve to injured nerve, 568 proteins were significantly upregulated and 471 were significantly downregulated. We used heatmaps to highlight GO pathways of interest related to nerve injury degeneration and regeneration. For our full dataset of raw data, please see the file at DOI: https://doi.org/10.13023/cbz6-ea76 which contains data for all of the proteins from our mass spectrometry analysis.

In the heatmaps, two criteria were used for significance when comparing naïve and injured nerve data: q-value ≤ 0.05 and Z ≥ |2| (log2). The effect size (Z-score) is the difference between the naïve and injured groups divided by the pooled standard deviation. Each individual cell in a heatmap shows the comparison between the naïve and injured nerve relative intensities for a particular protein in the participant. Red indicates significant increase, and blue significant decrease. Each column in the heatmap represents one participant (14 columns for n = 14 participants).

#### Growth factors

**Fig 5:**
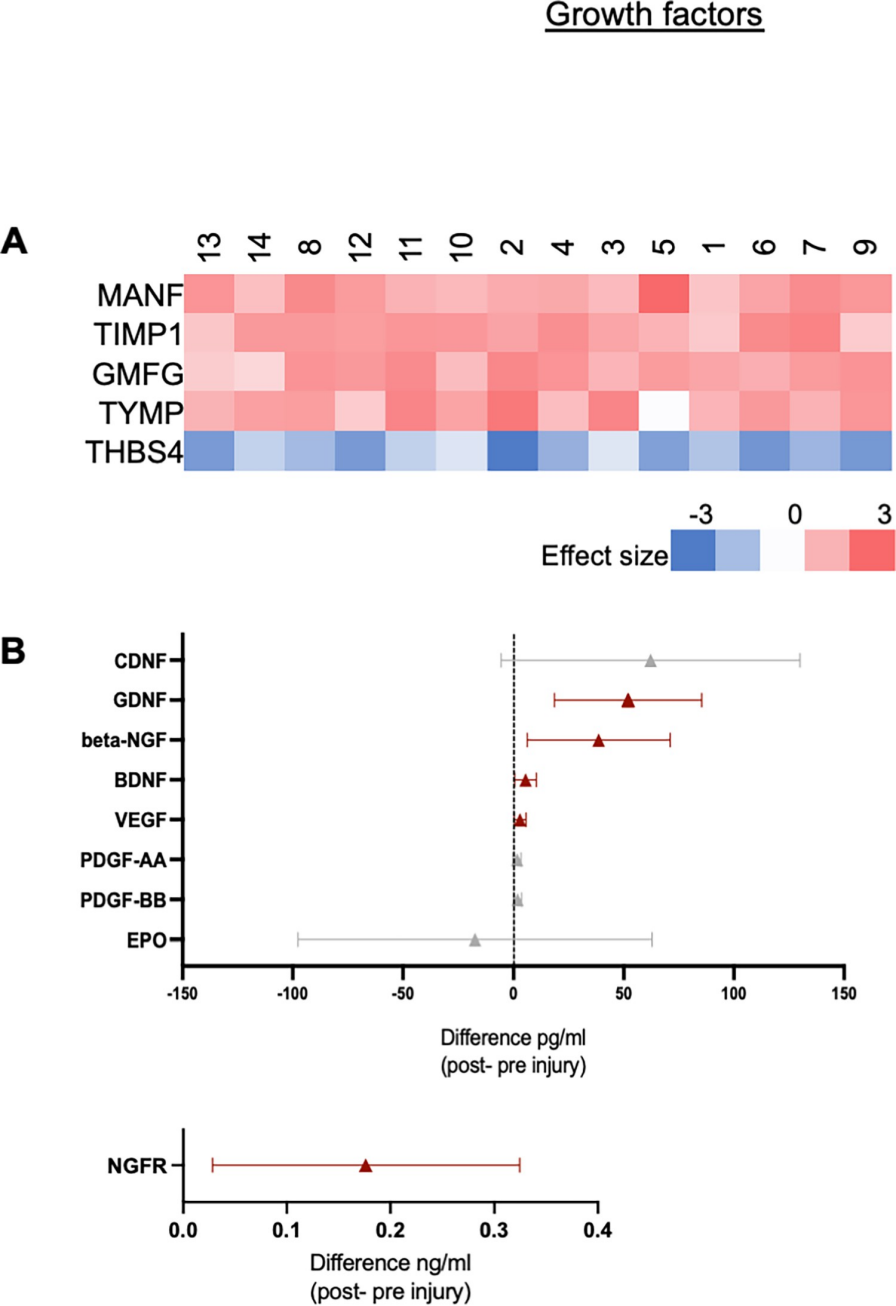
Changes in growth factors after injury based on the GO pathway “Growth Factor Activity”. **A.** Mass Spectrometry: Analysis of all the protein products that were significantly upregulated or downregulated (q-value ≤ 0.05 and Z ≥ |2|, range: -3 to 3). **B.** Immunoassay: Mean (triangle) differences, between naïve and injured tissue samples, and 95% CI for EPO, PDGF-AA and–BB, VEGF, BDNF, NGFR, beta-NGF, CDNF, and GDNF. Red markers represent mean difference CIs that were above zero and gray ones represent mean difference CIs that include zero.

Mass Spectrometry: Fig 5 shows all of the proteins that were significantly upregulated or downregulated (q-value ≤ 0.05 and Z ≥ |2| (log2)) from the GO pathway, Growth Factor Activity (GO:0008083). One protein was significantly downregulated after transection, and 4 proteins were significantly upregulated after injury (Fig 5). Out of 170 unique genes listed in this GO pathway, 25 genes were found in our data set, 5 of these proteins (20%, 5 of 25) were significantly differentially expressed when comparing naïve and injured tissue.

Immunoassay: Immunoassays were used to quantify the change in protein content of growth factors of interest that were not abundant enough to be detected via mass spectrometry. These factors of interest included EPO (mean difference: -17.37 pg/ml, n = 13), PDGF-AA (+1.58 pg/ml, n = 13) and PDGF–BB (+1.81 pg/ml, n = 15), VEGF (+2.96 pg/ml, n = 15), BDNF(+5.07 pg/ml, n = 15), NGFR (+0.18 ng/ml, n = 14), beta-NGF (+38.63 pg/ml n = 12), CDNF (+62.2 pg/ml n = 15), and GDNF (+51.97 pg/ml, n = 7), with the 95% confidence interval (CI) of the mean difference between injured and naïve tissues of PDGF-BB (-0.02, 3.65), PDGF-AA (-0.26, 3.42), EPO (-97.6, 62.9), VEGF (0.27, 5.66), BDNF (0.45, 9.68), CDNF (-5.6, 130.1), beta-NGF (6.19, 71.09), NGFR (0.03, 0.32), and GDNF (18.48, 85.45)(Fig 5B).

#### Myelination

**Fig 6:**
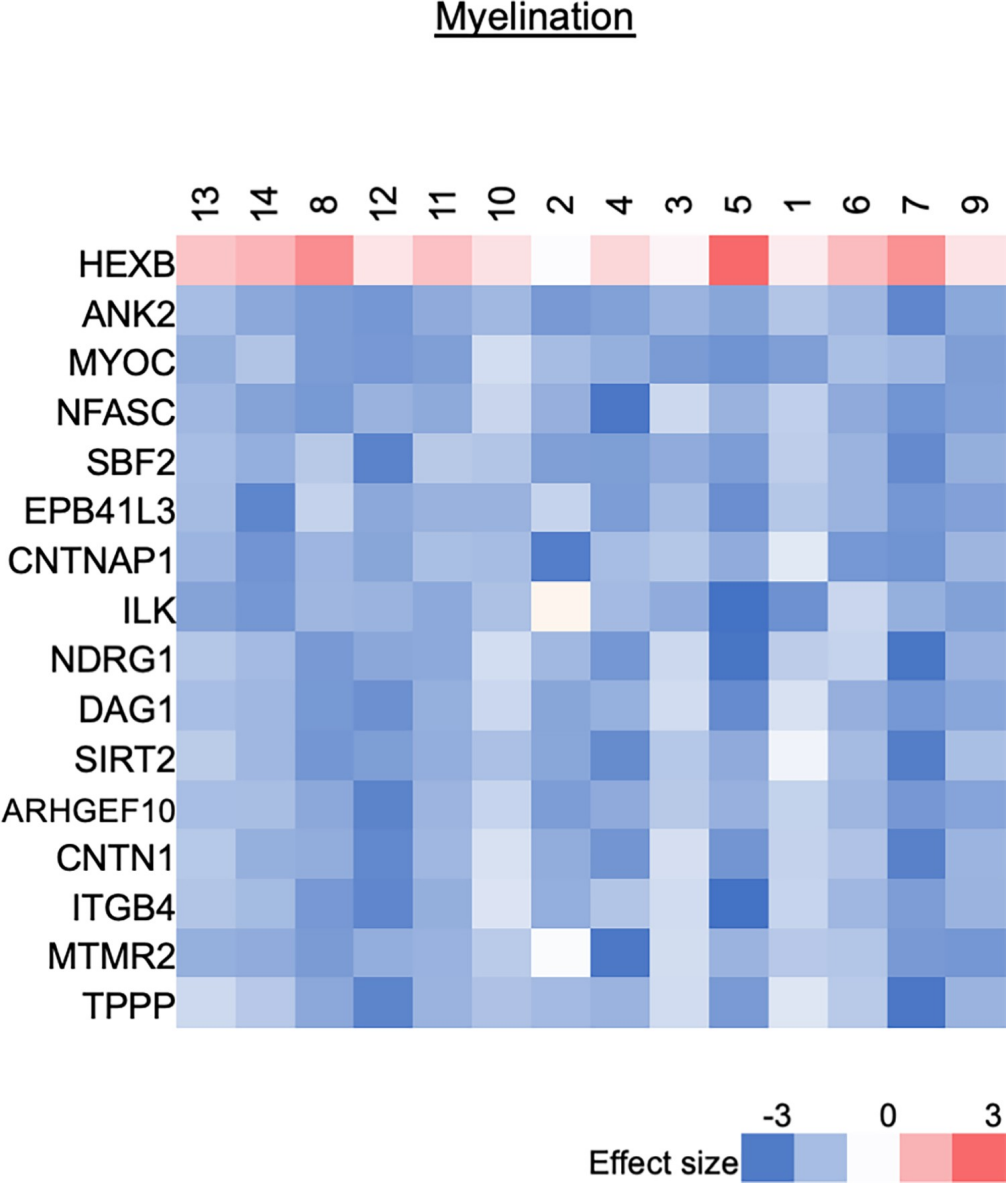
Changes in myelination after injury based on the GO pathway “Myelination”. Analysis of all the protein products that were significantly upregulated or downregulated from the GO pathway (q-value ≤ 0.05 and Z ≥ |2| (log2), range -3 to 3.

Mass Spectrometry: Fig 6 shows all of the protein products that were significantly upregulated or downregulated from the GO pathway “myelination” (GO: 0042552). Out of 116 unique genes listed in this GO pathway, 51 gene protein products were found in our data set, 15 proteins were significantly downregulated after transection (29% of 51), and 1 protein (2% of 51) were significantly upregulated after transection.

#### Schwann cell phenotypic changes

**Fig 7:**
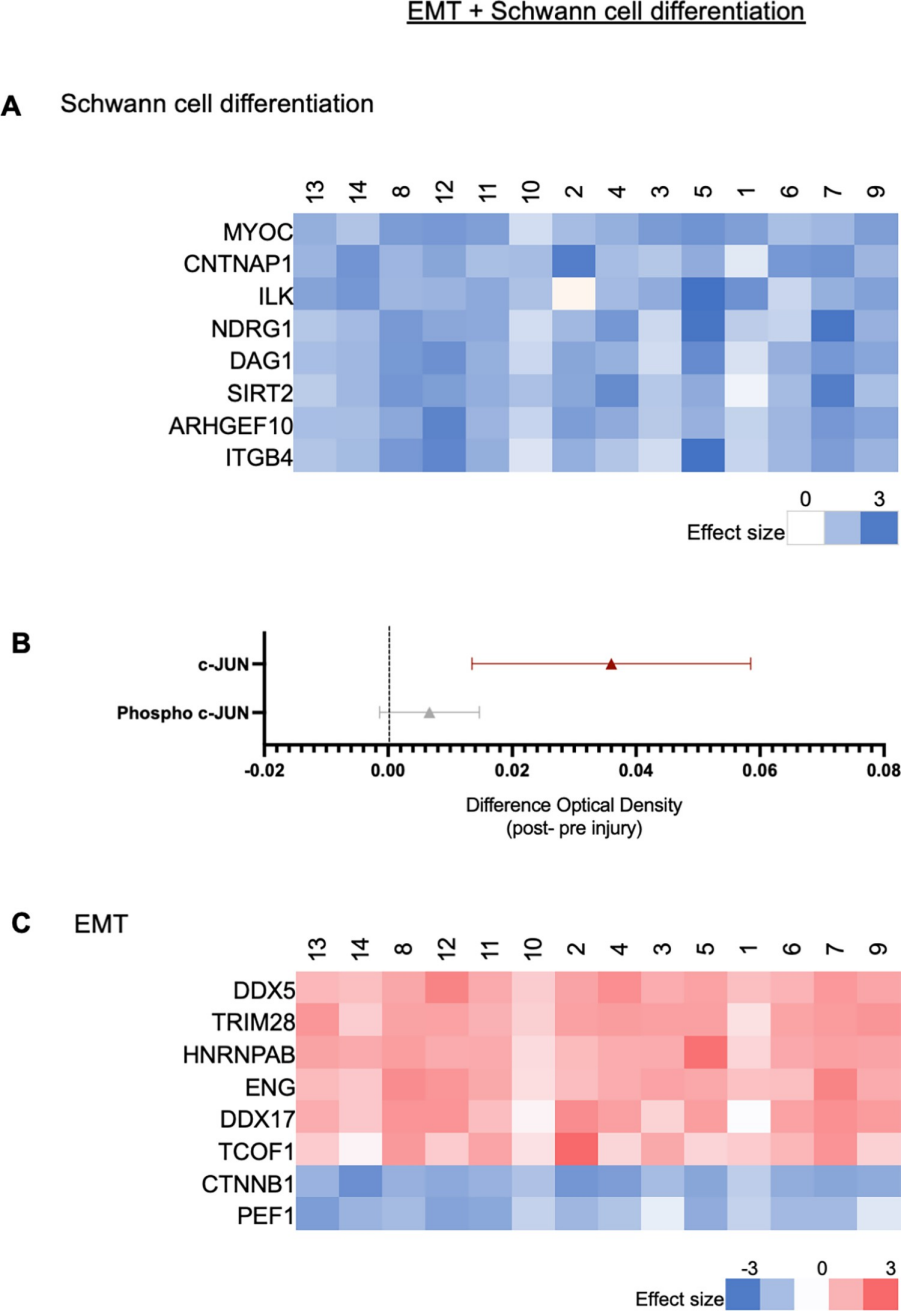
Changes in Schwann cell phenotype after injury based on the GO pathway “Schwann cell differentiation”. **A**. Mass spectrometry: All the proteins that were significantly upregulated or downregulated (q-value ≤ 0.05 and Z ≥ |2|, range -3 to 3). **B.** Immunoassay: The mean difference of c-JUN and its activated form phospho c-JUN content, in the naïve and injured tissue and 95% CI bands. Red markers represent mean difference CI that was above zero and gray one represent mean difference CI that included zero. **C.** Graph of mass spectrometry analysis of all the protein products that were significantly upregulated or downregulated from the GO pathway, “EMT”.

*Schwann cell differentiation*. Mass Spectrometry: Fig 7A shows all of the protein products that were significantly upregulated or downregulated from the GO pathway “Schwann cell differentiation” (GO: 0014037). Eight proteins were significantly downregulated after injury, and no proteins were significantly upregulated after injury. Out of 43 unique genes listed in this GO pathway, 20 gene protein products were found in our data set, 8 gene protein products (40% of 20) were significantly differentially expressed when comparing naïve to injured nerve.

Immunoassay: We quantified the content of total c-JUN (mean difference: +0.03 OD = 450, n = 15, CI: 0.02, 0.06) and its activated form phospho c-JUN (mean difference: +0.01 OD = 450, n = 15, CI: -0.0007, 0.0138) in the naïve and injured tissue (Fig 7A).

*Epithelial to Mesenchymal Transition (EMT)*. Mass Spectrometry: Fig 7B shows all of the protein products that were significantly upregulated or downregulated from the GO pathway “EMT” (0001837). Two proteins were significantly downregulated after injury, and 6 proteins were significantly upregulated after injury. Out of 85 unique genes listed in this GO pathway, 23 gene protein products were found in our data set, 8 gene protein products (35% of 23) were significantly differentially expressed when comparing naïve to injured nerve.

#### Anti-apoptosis factor changes

**Fig 8:**
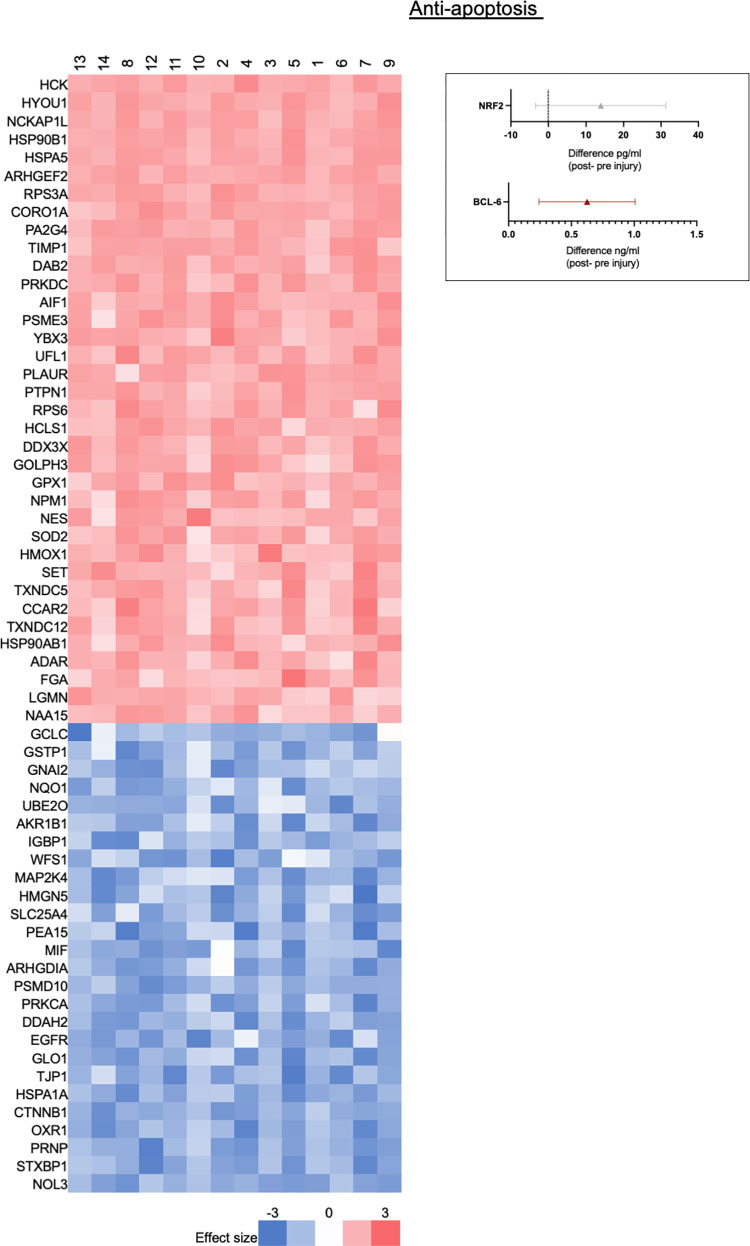
Anti-apoptosis factor changes after injury based on the GO pathway, “Negative Regulation of Apoptotic Processes”. Mass spectrometry analysis of all the protein products that were significantly upregulated or downregulated (q-value ≤ 0.05 and Z ≥ |2|, range -3 to 3). Immunoassay: Quantified NRF2 and BCL-6 changes after injury. The NRF2 graph (gray markers) shows the mean difference 95% CI that includes zero, and the BCL6 graph (red markers) represent mean difference 95% CI that is above zero.

Mass Spectrometry: Fig 8 shows all the protein products that were significantly upregulated or downregulated from the pathway “Negative Regulation of Apoptotic Processes” (GO: 0043066). Twenty-six proteins were significantly downregulated after injury, and 36 proteins were significantly upregulated after injury. Out of 928 unique genes listed in this GO pathway, 295 gene protein products were found in our data set, 62 gene protein products (21% of 295) were significantly differentially expressed when comparing naïve to injured nerve. Because we used samples from participants with PD, we particularly examined levels of α-synuclein (SNCA), a protein in this pathway, as aggregates of α-synuclein are associated with PD and deposits of phosporylated α-synuclein [38] are found in the sural nerve of people with PD. There was a significant downregulation of α-synuclein levels following injury based on a t-test (q ≤ 0.05) but with Z = 1.987, it failed to meet the Z ≥ |2| (log2) criterion.

Immunoassay: Additionally, we assayed for NRF2 (mean difference: +13.96 pg/ml, n = 14, CI: -3.39, 31.30) and BCL-6 (mean difference: +0.62 ng/ml, n = 15, CI: 0.24, 1.01) content where the 95% CI for BCL-6 was above zero in the injured tissue compared to naïve tissue (Fig 7).

#### Response to axonal injury

**Fig 9:**
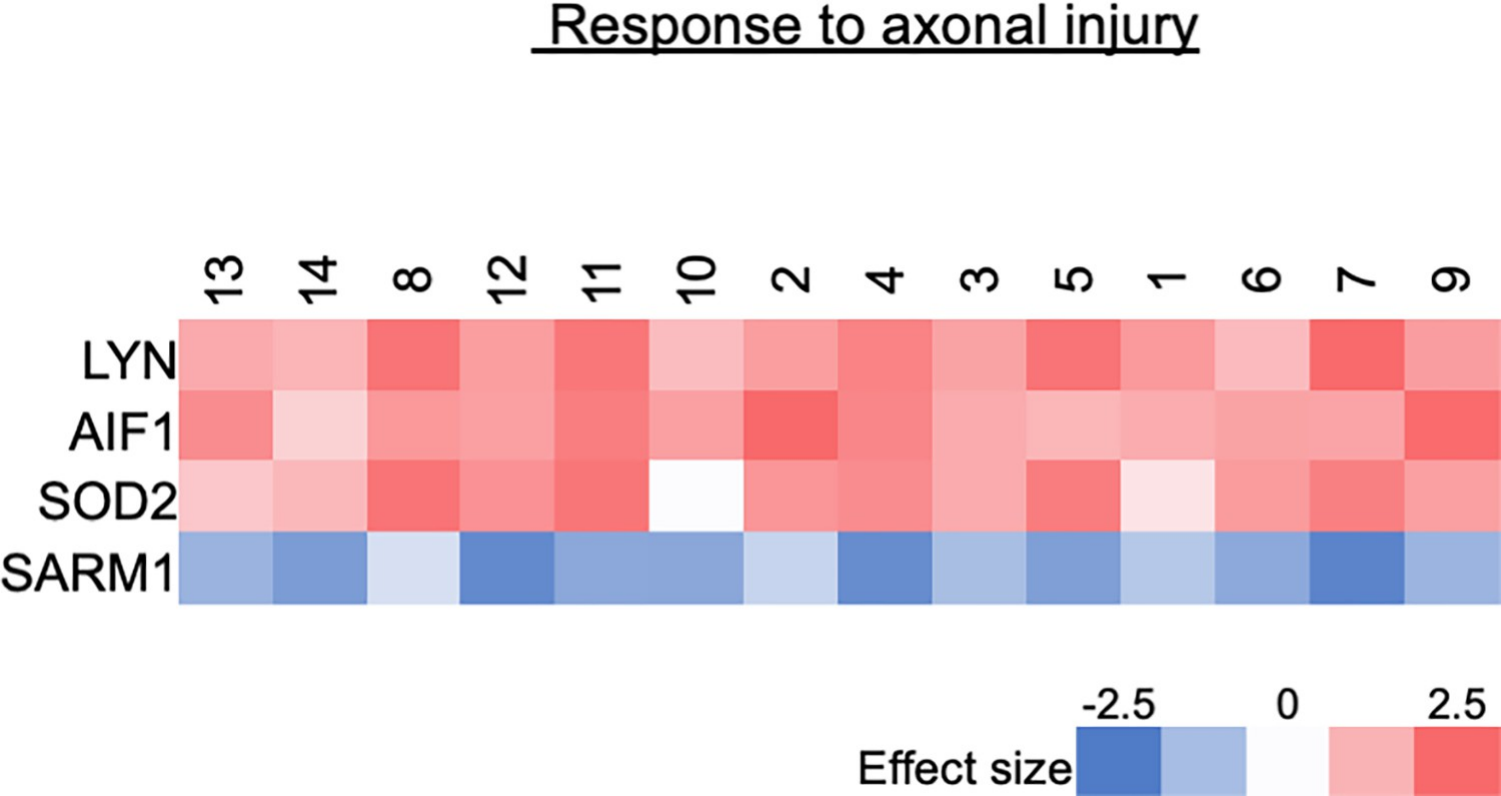
Axonal response after injury Graph of mass spectrometry analysis of all the protein products that were significantly upregulated or downregulated from the GO pathway, “Response to Axonal Injury” (q-value ≤ 0.05 and Z ≥ |2| (log2), range -2.5 to 2.5).

Mass Spectrometry: Fig 9A shows all of the protein products that were significantly upregulated or downregulated from the GO pathway, “response to axonal injury” (0048678). This is defined as **“**any process that results in a change in state or activity of a cell or an organism (in terms of movement, secretion, enzyme production, gene expression, etc.) as a result of an axon injury stimulus.” One protein was significantly downregulated after injury, and 3 proteins were significantly upregulated after injury. Out of 61 unique genes listed in this GO pathway, 29 gene protein products were found in our data set, 4 gene protein products (14% of 29) were significantly differentially expressed when comparing naïve to injured nerve.

## Discussion

### Clinical impact

#### Improving nerve regeneration

There are two main research approaches to improve repair and functional recovery: enhancing axonal regeneration and decreasing inflammation. One way to enhance axonal regeneration is through nerve growth factors. Translational researchers have isolated several of these neurotrophic factors and applied them to the proximal nerve stump or to bridging nerve conduits to enhance axonal regeneration in animal models [39,40]. Synthetic nerve guidance conduits have been infused with the neurotrophic factors, GDNF and NGF and slowly released to test axonal regeneration abilities across a 15mm gap in the rat sciatic nerve [41].

In our study, the growth factor profile of naïve and injured nerve could help us better determine how much to supplement nerve repair mechanisms with growth factors. Understanding the constituent levels could be especially important when implementing sustained or slow-release delivery systems to increase the growth factors at the injury. The results reported here focus on injury only at 2 weeks and serve as a starting point to provide context about human Schwann cell transformation, degeneration, and regeneration proteins. Even in this aged population of participants with PD, these regenerative processes are robustly upregulated after injury as seen in these analyses.

#### Using regenerating nerve as a neuroprotectant in neurodegenerative disease

In a departure from repairing the injured peripheral nerve itself, the regenerative capacity of the injured nerve can be harnessed as an implantable cell therapy. The regenerating tissue could serve as a vehicle for paracrine growth factors to neuroprotect unhealthy neurons. In a clinical trial, we are investigating the regenerating nerve as a possible treatment to neuroprotect degenerating substantia nigra neurons of people with PD [29,30]. Further, the upregulation of anti-apoptotic factors might aid in the cell survival of the implanted nerve tissue to tolerate the trauma of removal and implantation into a new site. The results presented here demonstrate the upregulation of growth and anti-apoptotic factors from the peripheral nerve tissues used in the trial.

### Study overview

The goal of this study was to determine how protein levels change after human peripheral nerve injury. The nerve transection was a necessary event for our clinical trial. Our group transected a peripheral nerve, activated degeneration and regeneration in the human sural nerve *in situ* at the ankle, and collected tissue samples from before and after injury, which would not have been ethically possible before this trial. Sural nerve biopsies are common neurosurgical procedures that can result in localized pain and paresthesias [42]. Participants in our studies reported these adverse events acutely but found them tolerable and not bothersome over time. The findings here provide an overview of the proteomic changes in peripheral nerve degeneration/regeneration that serve as a basis for targeted therapies to promote nerve regeneration and improve clinical outcomes.

### Growth factors after injury

The response to injury creates a neuroprotective microenvironment with the production of neurotrophic factors. Mass spectrometry and immunoassay results support this idea and aligns with previous findings that neurotrophic factors are higher after nerve injury [12,43–46]. Two weeks after injury, we observed higher mean levels in the injury samples compared to the naïve samples of growth factors: BDNF, CDNF, VEGF, GDNF, NGF, PDGF-AA and PDGF-BB (Fig 5B). These results align with our single nuclei RNA sequencing work in which the mRNA of these factors are present after peripheral nerve injury [47]. The protein-level profile of individual neuroprotective factors can vary greatly with time. After injury in mice, GDNF peaks around 7 days, and BDNF levels peak around 2–3 weeks [43]. Trophic factors involved in neurite outgrowth, cytoprotection, and neuronal survival such as mesencephalic astrocyte-derived neurotrophic factor (MANF) have also been reported to increase after peripheral nerve injury similar to the upregulation we observed in our analyses [48,49].

Other notable protein level increases from our findings include Glia Maturation Factor Gamma (GMFG), thymidine phosphorylase (TYMP), also known as platelet-derived endothelial cell growth factor, and tissue inhibitor of metalloproteinase-1 (TIMP1). GMFG was amongst the ligand mRNAs expressed in uninjured, 3- and 7- days post sciatic nerve injury using global transcriptomic analysis mRNA in a rodent model [50]. TIMPs play a major role in extracellular matrix (ECM) and tissue remodeling through its action as a potent inhibitor of ECM proteases such as matrix metallopeptidase 9 (MMP9). In a sciatic nerve axotomy model, *Timp1* was found to be the top 6^th^ gene that was induced [51]. Further supporting its role, mice with the *Mmp9* gene knocked out showed greater numbers of de-differentiated/immature myelinating Schwann cells in the injured nerve. This model also suggests that the *Mmp9*/*Timp1* axis guides myelinating Schwann cell differentiation and the molecular assembly of myelin domains during nerve regeneration [51].

### Myelination and Schwann cell phenotype after injury

Schwann cells play a major role in supporting peripheral nerves through myelination, maintaining axons, and guiding axons in regeneration after injury. Schwann cells from peripheral nerve tissue have a remarkable plasticity in that they rapidly adapt to injury through cellular transformation into a reparative phenotype in two stages. First, upon injury or transection, the transcription factor c-JUN is upregulated (Fig 7B), and Schwann cells de-differentiate from a myelinating phenotype into an immature phenotype, undergoing EMT in animal models and humans [28,45]. Second, Schwann cells transform into a reparative phenotype and release trophic factors and cytokines to support neuronal survival, and axon regeneration [12,43–45].

Our proteomic results support many of the processes reported in the literature in animal models including the initial downregulation of the myelinating phenotype [9]. We observed clear downregulation of myelination proteins such as neurofascin (NFASC), myotubularin-related protein 13 (SBF2), Erythrocyte Membrane Protein Band 4.1 Like 3 (EPB41L3, involved in myelin maintenance), and downregulation of proteins specific to the process of *peripheral* nerve myelination: integrin-linked protein kinase (ILK), dystroglycan 1 (DAG1), and sirtuin-2 (SIRT2)(Fig 6).

Classic Schwann cell markers in our mass spectrometry analysis such as S100B [52], MAG, MPZ, ERBB3, PLP1 all showed a significant downregulation by t-test (q ≤ 0.05), but failed to meet the Z ≥ |2| (log2) criterion (see raw data) suggesting in aggregate, a reduction in these markers. Our previous single nuclei RNA sequencing work supports our current data and demonstrates *MPZ*, *MBP*, and *MAG* downregulation two weeks after injury [47]. After the first step of degeneration which is clearing the myelin [9], Schwann cells then de-differentiate into more progenitor/stem cell-like phenotype. As such, we observed the upregulation of c-JUN (Fig 7B).

c-JUN, is a major transcription factor that activates the Schwann cell repair program by first downregulating the myelinating Schwann cell phenotype [11,12,43,44,53]. Both c-JUN and its activated form, phosphorylated c-JUN, were present more abundantly two weeks after injury than in the naïve tissue suggesting that at this timepoint, Schwann cell transformation into repair Schwann cells was actively occurring. Interestingly in our previous work using this transection paradigm [6], *JUN* mRNA levels were not detectable at two weeks suggesting that the peak for mRNA had diminished while the translated protein remained upregulated as evidenced by this current study.

c-Jun initiates Schwann cell de-differentiation into a progenitor-like state, and upregulates stem cell/progenitor factors such as Sox2, Notch1, Oct6 as a transition between two states. The Schwann cells undergo an EMT-like process in which cells become more like multipotent stem cells, and release neurotrophic factors to support cell survival [7]. We observed EMT proteins upregulation using mass spectrometry two weeks after injury. *JUN* is rarely expressed in normal, non-injured human nerve tissue [8]; however after injury, it was elevated in human nerve samples denervated for 4–50 days and up to 200 days [28]. In the early response to injury, in many tissues, activation of EMT and stemness is associated with increased cell motility, proliferation, phenotypic flexibility, tissue remodeling and differentiation flexibility [54,55], all which are important in repair and neuroprotection.

Schwann cells transform from the myelinating phenotype to a reparative phenotype via EMT mechanisms [28,45]. Single nuclei RNA sequencing demonstrates the presence of a repair Schwann cell marker, *NGFR* upregulated after injury [47]. Additionally, the EMT GO pathway (GO: 0001837) includes two DEAD-Box Helicase 5 (DDX) genes including DEAD-Box Helicase 5 (DDX5, also known as p68) and DEAD-Box Helicase 17 (DDX17) which are upregulated after injury. DDX proteins are RNA helicases involved in altering RNA secondary structures such as in translation initiation, coregulating transcription, and regulating splicing [56]. In the central nervous system, DDX5 has shown to complex with MBP in immature/progenitor oligodendrocytes and is involved in the post-transcriptional regulation of MBP protein synthesis, an alternative splicing of MBP, which ultimately affects the expression of MBP [56].

#### Schwann cell vs. axonal protein distinction

To distinguish between Schwann cells vs. axonal protein changes after injury, the results shown in Fig 7A reflect Schwann cell-specific changes (GO: 0014037, “Schwann cell differentiation”), and axonal-specific changes are reflected in Fig 9 for response to axonal injury (GO: 0048678, “Response to axonal injury”). In the global analysis with functional annotation (Tables 3 and 4), we found that the top downregulated GO pathways related more to cytoskeletal structure, neurons and synapses compared to Schwann cells which suggests that at 2 weeks, neuronal downregulation pathways dominated over Schwann and myelin changes likely a reflection of Wallerian degeneration.

Nerve sub-compartments to the injury/regenerative process:

Of note, the GO heatmaps highlighted here do not distinguish between the different nerve sub-compartments, such as the perineurium or endoneurium; however inspecting the raw mass spectrometry data reveals some changes in sub-compartment cell markers (see mass spectrometry data file, DOI: https://doi.org/10.13023/cbz6-ea76). Perineurial protein markers, solute carrier family 2 member 1 (SLC2A1) and LIM Domain 7 (LMO7) were significantly downregulated (q-value ≤ 0.05 and Z ≥ |2| (log2)) after peripheral nerve injury in the mass spectrometry data (cell type markers from [57]). Other sub-compartment markers, such as those for the endoneurium and epineurium, were not captured in the proteomic analyses because they were not sufficiently detected above background. Here we also observed changes in cell-type markers related to the regenerative process that we previously observed through RNA sequencing, significant upregulation such as mesenchymal cell (*PDGFRA*, *THY1*, *ADAM12*, *and TWIST1*) endothelial cell (*PECAM*, *VWF*) [6] and proliferating markers (*FOXM1*) (data set [6]).

### Data interpretation

Important limitations apply to the interpretation of these results. We used naïve and injured peripheral nerve tissue from participants with PD which may differ from non-PD tissue. Synucleinopathies are characteristic in people with PD in both their central and peripheral nervous systems. α-synuclein aggregates have been found in the sciatic nerves and pharyngeal nerves of post-mortem patients with PD [58]. Of note, our results from mass spectrometry show that the α-synuclein protein showed a downregulation trend after transection injury (q-value ≤ 0.05, Z = -1.99). Notably, the transection did not cause further upregulation of α-synuclein. Furthermore, as people with PD have a higher incidence of neuropathy [59,60], we recognize that using tissue from participants with PD introduces the concern of neuropathies. The presence of a neuropathy was not an exclusionary criterion for the underlying clinical trial. Most participants in the trial did not report a history of neuropathy.

As in Welleford et al. [6], the design of the underlying clinical trial necessitated a delay in flash freezing the injury samples which could affect these protein profile results. However, the direction of changes in protein levels, after injury, in the GO pathways of myelination, EMT, and axonal regeneration suggest that elements of the proteome in the injured state are in concordance to reported changes in animal studies [7,20,21,43]. The underlying clinical trial design from where these samples were obtained limited our ability to adequately control for the existence of neuropathy, comorbidities that might influence regeneration, age, disease duration, genetics of participants or even the sample freezing time differences among individuals; thus, for interpreting the analyses, we quantified the degree of similarity among different samples by calculating Lin’s CCC (Fig 4B). We found a strong homogeneity among samples within the naïve and injured groups. Therefore, even with these limitations, we believe that this study provides insight on the proteomic profile of degenerating/regenerative nerves two weeks after an injury and contributes a database of useful information for establishing biomarkers of peripheral nerve injury in humans.

**Summary**

○ We present a proteomic picture of the degenerative and regenerative processes in humans before and after peripheral nerve injury *in situ*.
○ We provide a database for peripheral nerve repair proteins which may support clinical translation into supplementing growth factors at the nerve injury site.
○ Our results support many processes reported in animal models—down-regulation of myelination, Schwann cell de-differentiation, and upregulation of growth factors.
